# Evaluation of post-dilatation on longitudinal stent deformation and postprocedural stent malapposition in the left main artery by optical coherence tomography (OCT): an in vitro study

**DOI:** 10.1186/s12880-024-01223-6

**Published:** 2024-03-01

**Authors:** Qing He, Yuqi Fan, Zuojun Xu, Junfeng Zhang

**Affiliations:** Department of Cardiology, Shanghai Ninth People’s Hospital, Shanghai Jiaotong University School of Medicine, 639 Zhizaoju Road, Shanghai, 200011 China

**Keywords:** Optical coherence tomography, Vessel, Longitudinal stent deformation, Stent malapposition

## Abstract

**Background:**

The diameter of the ostial and proximal left main coronary artery can be greater than 5.0 mm. However, the diameters of the mostly available coronary drug-eluting stents (DESs) are ≤ 4.0 mm. Whether high-pressure dilatation can increase the diameter of stents from 4.0 to 5.0 mm and whether post-dilatation leads to longitudinal stent deformation (LSD) of 4.0-mm-diameter stents have rarely been studied. Therefore, this study aims to evaluate LSD and stent malapposition of six types of commercially available 4.0-mm-diameter stents in China in a 5.0-mm-diameter artificial blood vessel model by optical coherence tomography (OCT) in vitro.

**Methods:**

The left main coronary artery was simulated by a truncated cone-shaped silicone tube. The internal diameters were 4.0 mm at one end of the silicone tube and 5.0 mm at the other end. Six different types of coronary stents widely used in China were selected for this study. Each stent was respectively implanted into the simulated blood vessel and dilated to a diameter of 4.2 mm according to the stent-balloon pressure compliance table. The stents were subjected to post-dilatation with a 5.0 × 15-mm noncompliant balloon. The LSD ratio of the longitudinal axis of each stent and stent malapposition were measured through OCT, and any fractures of the stents were determined.

**Results:**

None of the six types of stents fractured following post-dilatation. The longitudinal axes of the BuMA and Excrossal stents were slightly shortened, while the other stents were elongated after high-pressure post-dilatation. All stents expanded to a diameter of 5.0 mm without incomplete stent apposition, except for the Nano Plus stent, which remained malapposed after high-pressure post-dilatation.

**Conclusion:**

All 4.0-mm-diameter stents can be expanded to a diameter of 5.0 mm by noncompliant balloon post-dilatation without stent strut fracture. Most stents were found to be well apposed after high-pressure post-dilatation. However, LSD was observed after post-balloon dilatation. Stent malapposition might be positively correlated with the percentage change in stent length.

**Supplementary Information:**

The online version contains supplementary material available at 10.1186/s12880-024-01223-6.

## Introduction

Coronary intervention guidelines state that the effect of percutaneous coronary intervention (PCI) using a drug-eluting stent (DES) for anatomically appropriate left main coronary artery lesions with a SYNTAX score ≤ 22 is equivalent to that of coronary artery bypass surgery [1]. The diameter of the ostial and proximal left main coronary artery is usually larger than 5.0 mm. However, the diameters of the coronary DESs commonly used in clinical practice are ≤ 4.0 mm. Few studies have investigated whether high-pressure dilatation can increase the diameter of a stent to greater than 5.0 mm, and blood vessel models were not used in these studies [2, 3]. Additionally, dilatation of stents with high-pressure noncompliant balloons with a diameter exceeding the indicated diameter of the stent may lead to shortening of the stent, but relevant data are lacking. Among the current intraluminal imaging technologies available, optical coherence tomography (OCT) offers the highest amount of detail to visualize the vessel wall morphology, with an axial resolution of 10–20 μm. OCT can finely detect normal arterial walls and plaque morphology, accurately measure the stent length, diameter, and lumen area, and accurately evaluate stent deployment [4]. Therefore, this study aimed to investigate LSD and stent malapposition of six types of 4.0-mm-diameter DESs by OCT using a 5.0-mm-diameter artificial vessel model following high-pressure post-dilatation.

## Methods

### In vitro Vessel Model, Stent Implantation and Dilatation, and OCT examination

**Fig. 1:**
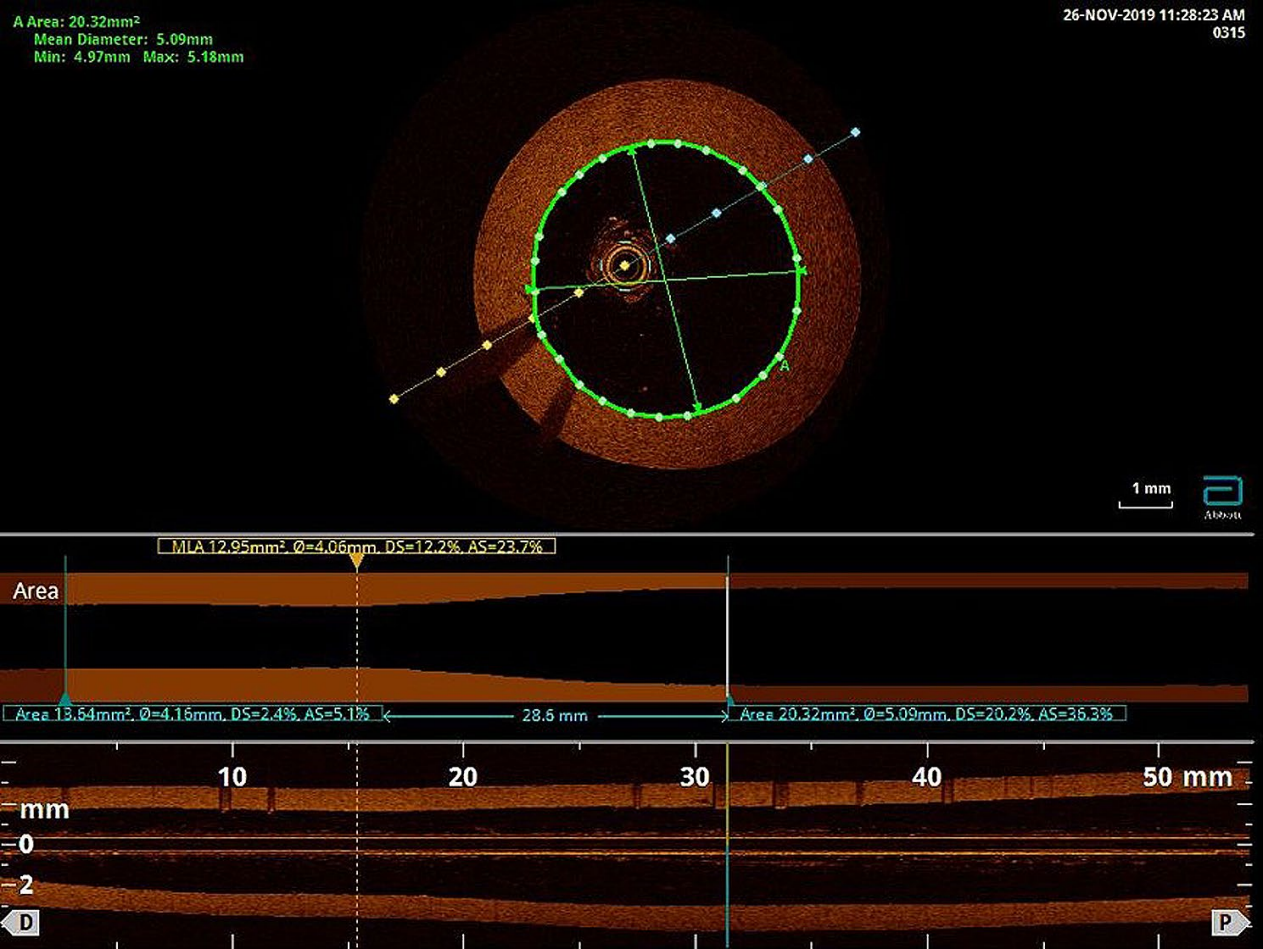
Characteristics of the silicon vessel models used in this study. A truncated cone-shaped silicone tube was used to model the left main coronary artery. A 2.7 Fr C7 Dragonfly OCT imaging catheter (Abbott) was used to measure the size of the silicone tube; the distal segment was approximately 20 mm long, and the internal diameter was 4.16 mm. The diameter expanded to 5.09 mm at the proximal segment, which had a length of approximately 25 mm. The material of the silicon model reflects the actual vessel frictional resistance and hardness

A truncated cone-shaped silicone tube was used to model the left main coronary artery, with the smaller-diameter end mimicking the distal segment and the larger-diameter end mimicking the entry of the artery [5]. A 2.7 Fr C7 Dragonfly OCT imaging catheter (Abbott Vascular, Santa Clara, CA, USA) was used to accurately measure the size of the silicone tube; the distal segment was approximately 20 mm long, and the internal diameter was 4.16 mm. The diameter expanded to 5.09 mm at the proximal segment, which had a length of approximately 25 mm (Fig. 1). The OCT pullback speed was 180 Frames/Sec. We studied six types of coronary stents widely used in clinical practice in China, including the XIENCE Xpedition 4.0 mm × 28 mm (Abbott Vascular, Santa Clara, CA, USA), the Helios 4.0 mm × 28 mm (Kinhely, Shenzhen, China), the Firehawk 4.0 mm × 29 mm (MicroPort, Shanghai, China), the BuMA 4.0 mm × 30 mm (SINOMED, Tianjin, China), the Nano Plus 4.0 mm × 29 mm (Lepu medical, Beijing, China), and the Excrossal 4.0 mm × 29 mm (JWMS, Weihai, China). The key characteristics of each DES are shown in Supplemental Table S1. Each stent was deployed into the simulated blood vessel and expanded to a diameter of 4.2 mm according to the stent-balloon pressure compliance table. Then, each stent was subjected to post-dilatation with a 5.0 × 15-mm noncompliant balloon from the distal to the proximal segments at pressures of 6 atm, 12 atm and 20 atm, and the duration of each dilatation was 20 s. Each type of stent was subjected to three independent tests.

### Data Analysis

Each stent was subjected to an initial OCT examination after being deployed, and the following parameters were measured: stent length 1 (SL1), the lumen area at the proximal end of the stent (lumen area 1, LA1), and the stent area (stent area 1, SA1). The stent malapposition rate before post-dilatation was calculated according to the following formula: stent/lumen area rate 1 (SLAR1) = (LA1-SA1)/LA1 × 100%. Subsequently, each stent was subjected to a second OCT examination after post-dilatation by a 5.0-mm noncompliant balloon that measured stent length 2 (SL2), the lumen area at the proximal end of the stent (lumen area 2, LA2), and the stent area (stent area 2, SA2). The LSD rate after post-dilatation was calculated by the following formula: LSD rate = (SL2-SL1)/SL1 × 100%. The stent malapposition rate after post-dilatation was calculated by the following formula: stent/lumen area rate 2 (SLAR2) = (LA2-SA2)/LA2 × 100%. Any fractures in the stents were identified.

### Statistical analysis

The data were analyzed and processed with GraphPad Prism software (version 8.4). The normally distributed quantitative data are presented as the mean ± standard deviation ($\stackrel{-}{\text{x}}\pm \text{s}$) and were subjected to one-way ANOVA followed by a post hoc Tukey’s test for comparisons between two groups. All tests were two-tailed. Differences with a *p*-value < 0.05 were considered statistically significant.

## Results

### LSD rates of different types of 4.0-mm-diameter stents following post-dilatation with a 5.0-mm noncompliant balloon

**Fig. 2:**
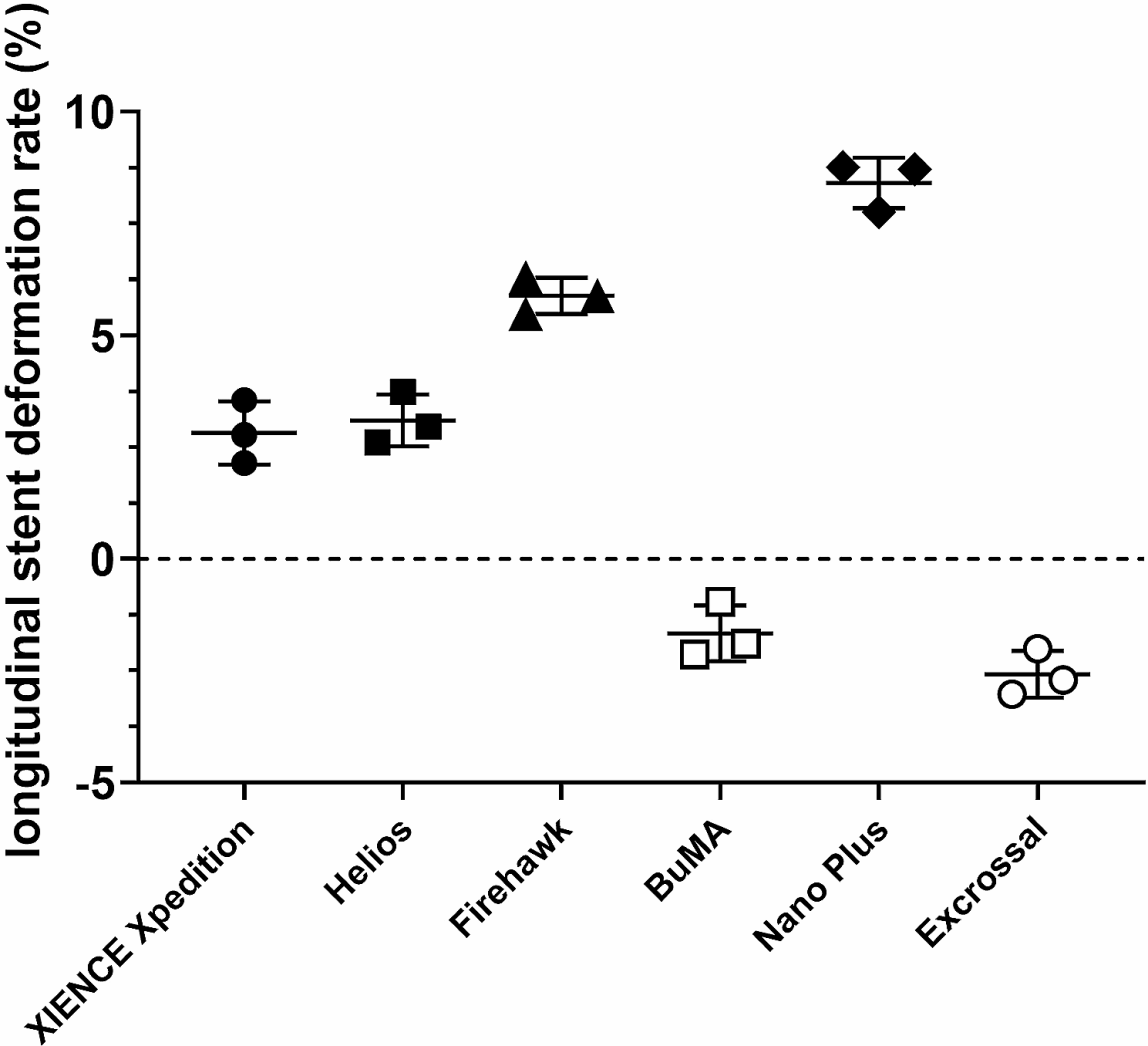
Longitudinal stent deformation (LSD) rates of different types of 4.0-mm-diameter stents following post-dilatation with a 5.0-mm noncompliant balloon. Six types of stents including the XIENCE Xpedition 4.0 mm × 28 mm, the Helios 4.0 mm × 28 mm, the Firehawk 4.0 mm × 29 mm, the BuMA 4.0 mm × 30 mm, the Nano Plus 4.0 mm × 29 mm, and the Excrossal 4.0 mm × 29 mm were subjected to an initial OCT examination after being deployed. Stent length 1 (SL1) of each stent was measured. Subsequently, each stent was subjected to a second OCT examination after post-dilatation by a 5.0-mm noncompliant balloon that measured stent length 2 (SL2). The LSD rate post-dilatation was calculated with the following formula: LSD rate = (SL2-SL1)/SL1 × 100%. The XIENCE Xpedition, Helios, Firehawk, and Nano Plus stents were slightly elongated in the longitudinal axis after high-pressure dilatation, and their average elongation rates were 2.82%, 3.09%, 5.88%, and 8.41%, respectively. The BuMA and Excrossal stents were slightly shortened in the longitudinal axis after high-pressure dilatation, and their average shortening rates were − 1.67% and − 2.58%, respectively (*P* < 0.05, *n* = 3)

Fractures were not detected in all six types of stents. The XIENCE Xpedition and Helios stents were slightly elongated in the longitudinal axes after high-pressure dilatation, and their average LSD rates were 2.82% and 3.09%, respectively. However, the LSD rates of the Firehawk and Nano Plus stents were greater than 5.0%, and their average LSD rates were 5.88% and 8.41%, respectively. The BuMA and Excrossal stents were slightly shortened in the longitudinal axes after high-pressure dilatation, and their average LSD rates were − 1.67% and − 2.58%, respectively (Fig. 2).

### Stent apposition rates of different types of 4.0-mm-diameter stents following post-dilatation by a 5.0-mm high-pressure balloon

**Fig. 3:**
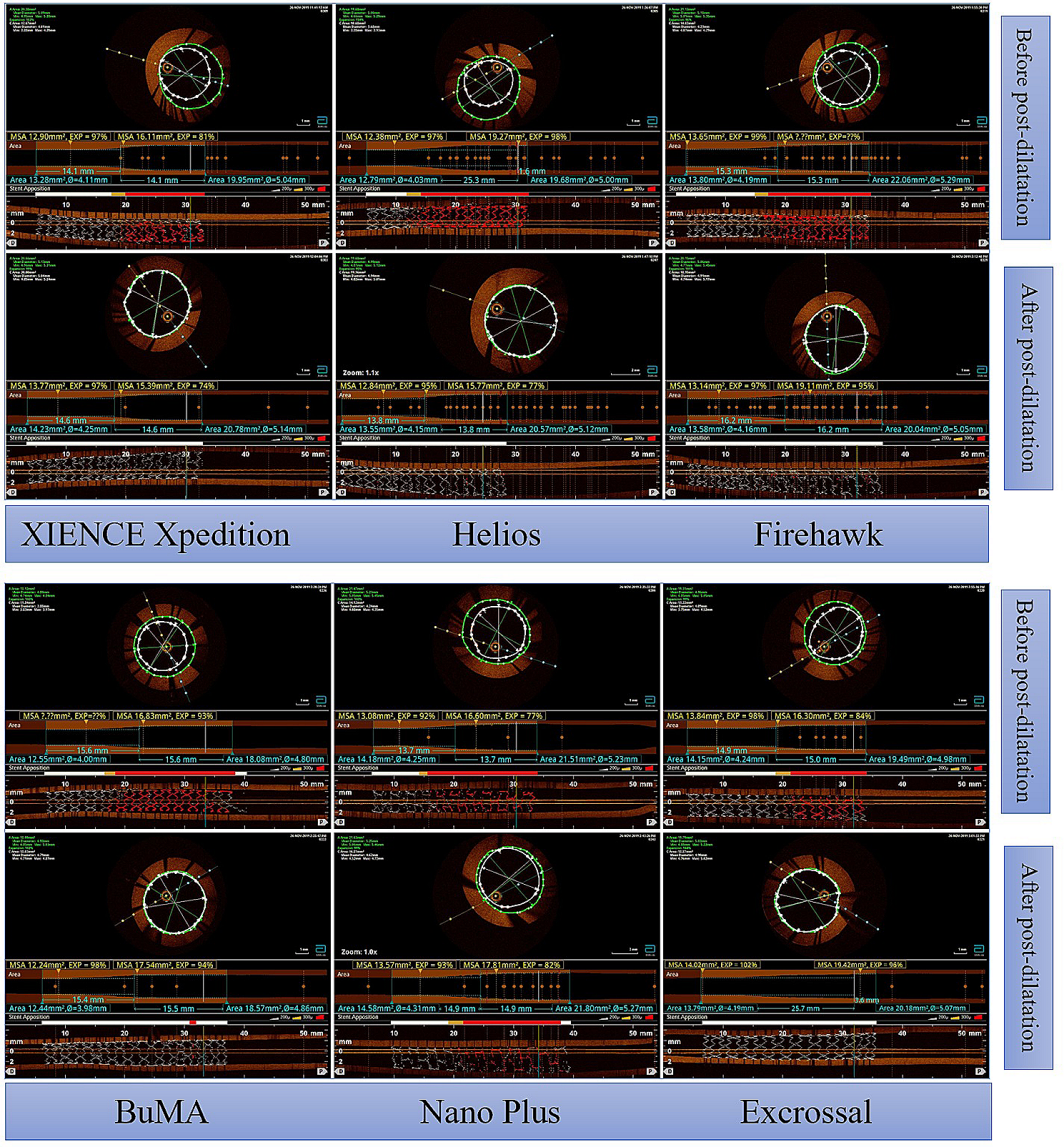
OCT findings of different types of 4.0-mm-diameter stents following post-dilatation by a 5.0-mm high-pressure balloon. The XIENCE Xpedition 4.0 mm × 28 mm, the Helios 4.0 mm × 28 mm, the Firehawk 4.0 mm × 29 mm, the BuMA 4.0 mm × 30 mm, the Nano Plus 4.0 mm × 29 mm, and the Excrossal 4.0 mm × 29 mm were subjected to OCT examination before and after post-dilatation with a 5.0-mm noncompliant balloon

**Fig. 4:**
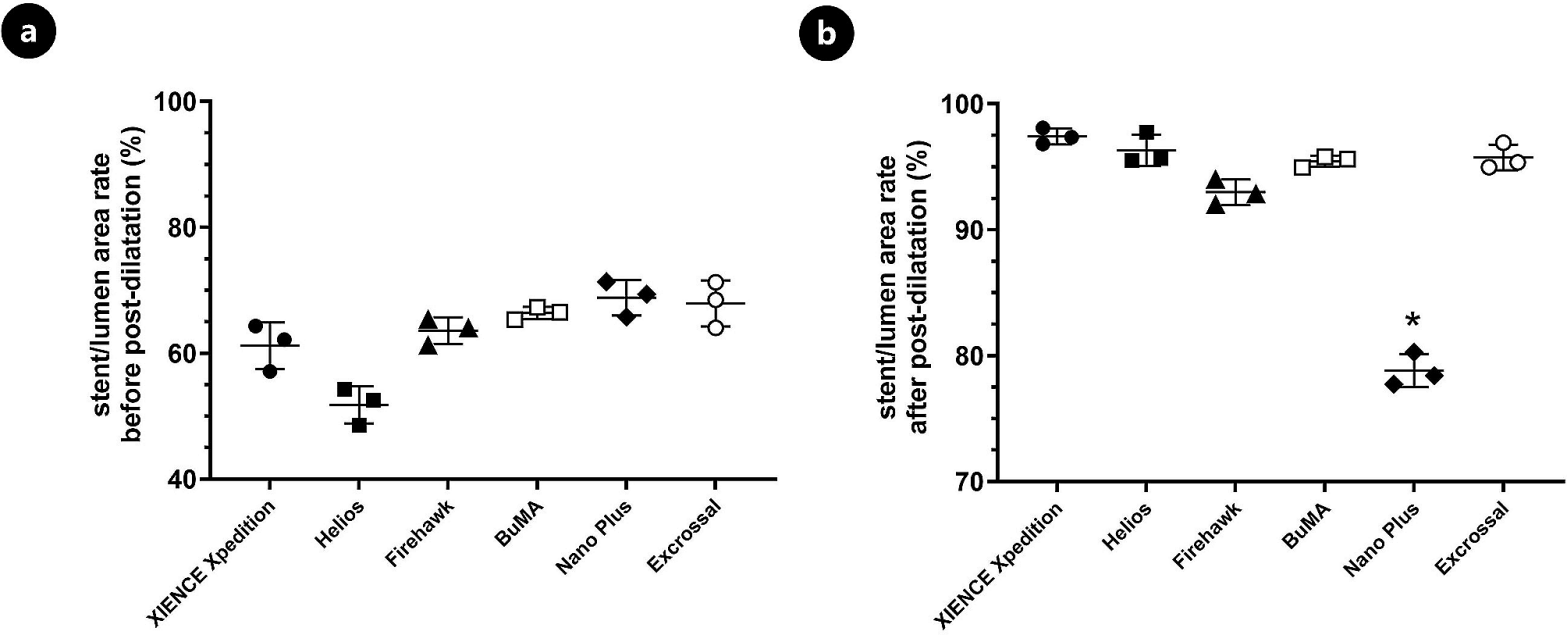
Stent apposition rates of different types of 4.0-mm-diameter stents following post-dilatation by a 5.0-mm high-pressure balloon. The lumen area at the proximal end of the stent (lumen area 1, LA1), the stent area (stent area 1, SA1), and the stent/lumen area rate [SLAR1 = (LA1-SA1)/LA1 × 100%] before post-dilatation were measured and calculated. Subsequently, the lumen area at the proximal end of the stent (lumen area 2, LA2), the stent area (stent area 2, SA2), and the stent/lumen area rate [SLAR2 = (LA2-SA2)/LA2 × 100%] post-dilatation were obtained. Following post-dilatation, the apposition rates of the XIENCE Xpedition, Helios, Firehawk, BuMA, and Excrossal stents were significantly improved, and their average apposition rates were 97.42%, 96.31%, 92.98%, 95.45, and 95.75%, respectively. However, the Nano Plus stent apposition rate was only 78.80% after high-pressure dilatation (*P* < 0.05, *n* = 3)

After the expansion of each 4.0-mm-diameter stent to a diameter of 4.2 mm according to the stent-balloon pressure compliance table, stent malapposition of the proximal end was examined by OCT, and the average apposition rates of the six types of stents were 61.20%, 51.77%, 63.60%, 66.39%, 68.83%, and 67.91%. Following post-dilatation by a 5.0-mm high-pressure balloon, the apposition rates of the XIENCE Xpedition, Helios, Firehawk, BuMA, and Excrossal stents were significantly improved, with average apposition rates of 97.42%, 96.31%, 92.98%, 95.45, and 95.75%, respectively. The Nano Plus stent was still apposed incompletely after high-pressure dilatation, with an apposition rate of 78.80% (Figs. 3 and 4).

## Discussion

The diameter of the ostial and proximal left main coronary artery is usually larger than 5.0 mm. However, there are no > 4.0-mm-diameter coronary DESs available in China and some other countries till now. Our test results show that none of the six types of 4.0-mm-diameter DESs were fractured after high-pressure dilatation with a 5.0-mm noncompliant balloon and that LSD was caused by post-dilatation after stent implantation. While the Nano Plus stent was apposed incompletely after post-dilatation, the other stents were dilatated to a diameter of 5.0 mm and were well apposed.

Two previous studies showed that 4.0-mm-diameter stents could be dilatated to a diameter of 5 mm or higher [2, 3]. However, these studies did not include tests using an in vitro blood vessel model and therefore could not simulate the actual blood vessels. Additionally, the observations and measurements were obtained by three-dimensional reconstructions using optical microscopy and Micro-CT imaging. In contrast, we used a truncated cone-shaped silicone tube to model the left main coronary artery, which is closer to the actual condition of the blood vessel. Furthermore, we observed the lumen by OCT, which is more comparable to the in vivo situation, and the measurements of the changes in the longitudinal axis and the apposition rates of the stents are more accurate and intuitive. Regarding left main coronary lesions, a certain risk exists when assessing the severity of stenosis only through angiography. Current myocardial revascularization guidelines recommend interventional therapy under the guidance of intravascular imaging for left main lesions, which is conducive to optimization of stent implantation to ensure sufficient apposition of the stent [6]. A previous in vitro OCT study suggested that stent malapposition is the main cause of stent thrombosis [7]. Therefore, good stent apposition is extremely important in the treatment of ostial left main coronary lesions. Our result revealed that stent malapposition might be positively correlated with the percentage change in stent length (the LSD rate). LSD, which is defined as distortion of a stent in the longitudinal axis, has been recognized as an important complication during PCI procedures [8]. Various risk factors have been identified for LSD, such as lesion complexity, stent design, post-dilatation, and supporting devices. Apart from the factors related to lesion characteristics and the complexity of the procedures, stent material and design play vital roles in stent longitudinal integrity. The connectors which join the adjacent stent rings provide longitudinal support for the stent. The fewer connectors attached to stent rings, the larger open cells the stents have. Compared with stents with more than two connectors, less force was required to compress or elongate stents with two connectors [9]. LSD may be difficult to detect angiographically, and the use of OCT or intravascular ultrasound (IVUS) can help confirm the diagnosis and guide treatment decisions, as well as avoid complications such as stent thrombosis [10]. In our current study, we showed that the LSD rates of the Firehawk and Nano Plus stents were greater than the other stents by OCT examination. The higher LSD in these two stents could be due to relatively few connectors compared with other stent platforms. Previous studies suggested that the proximal optimization technique (POT) and post-dilatation from the proximal site of the stent were useful for reducing the incidence of LSD [8, 11].

Stent malapposition can result in short- or long-term adverse clinical events. The material and design of the stent and post-dilatation strategies affect acute recoil and stent expansion. 316 L stainless steel, open-cell design and stent with thin struts are likely to cause low radial strength, subsequently leading to stent malapposition. Moreover, the number of the connectors between loops and the orientation of these connectors also contribute to radial support. Post-dilatation using large non-compliant balloon is usually performed to overcome the malapposition. This is extremely important for complex coronary lesions, such as left main lesions, severe calcification and bifurcation lesions. A strategy of several short post-dilatations with non-compliant balloon was better than a single long inflation for improving the recoil and enlarging the stent area. However, no single stent design is suitable for all variety of CAD and improvement in one aspect of a stent will compromise another aspect. Understanding this tradeoff and the mechanical properties of each stent design may help interventional cardiologists in individualized device selection and facilitate procedural success.

## Conclusion

In summary, 4.0-mm-diameter stents can be expanded to a diameter of 5.0 mm by high-pressure post-dilatation. LSD was observed after post-dilatation in a truncated cone-shaped vessel model. Stent malapposition might be positively correlated with the percentage change in stent length. Upon stent implantation for left main coronary lesions, the stent should be sufficiently post-dilatated using the POT under the guidance of intravascular imaging to reduce the risk of LSD and stent malapposition.

### Electronic Supplementary Material

Below is the link to the electronic supplementary material.

Supplementary Material 1

## Data Availability

The data used to support the findings of this study are included within the article.
