# Supplementary material for: Evaluation of post-dilatation on longitudinal stent deformation and postprocedural stent malapposition in the left main artery by optical coherence tomography (OCT): an in vitro study
Source: BMC Med Imaging. 2024 Mar 1;24:53. doi: 10.1186/s12880-024-01223-6 (PMC10908124; doi:10.1186/s12880-024-01223-6)
Supplement: Supplementary file 1 — Supplementary Material 1 [file 12880_2024_1223_MOESM1_ESM.docx]

**Supplementary Material**

**Table S1. Key characteristics of each stent**

| Samples | Stent design family | Material | Strut thickness  (μm) |
| --- | --- | --- | --- |
| XIENCE Xpedition | **In-phase,**  **peak-to-peak** | **Cobalt chromium** | **81** |
| Helios | **Out-of-phase, peak-to-valley** | **Cobalt chromium** | **90** |
| Firehawk | **In-phase,**  **peak-to-valley** | **Cobalt chromium** | **91** |
| BuMA | **Out-of-phase,** **peak-to-valley** | **316 L stainless steel** | **100** |
| Nano Plus | **Out-of-phase, peak-to-valley** | **316 L stainless steel** | **100** |
| Excrossal | **Out-of-phase,**  **peak-to-valley** | **Cobalt chromium** | **84** |
